# Assessing the clinical efficacy of phacoemulsification cataract extraction in treating acute primary angle closure and fellow primary angle closure suspect eyes using AS-OCT

**DOI:** 10.3389/fmed.2024.1436991

**Published:** 2024-09-24

**Authors:** Rumin Zhao, Wenhui Geng, Yunlong Wu, Zijian Zhang, Bojun Zhao

**Affiliations:** ^1^Department of Glaucoma, Shandong Lunan Eye Hospital, Linyi, Shandong Province, China; ^2^Department of Ophthalmology, Shandong Provincial Hospital Affiliated to Shandong First Medical University, Jinan, China

**Keywords:** glaucoma, phacoemulsification, primary angle-closure disease, primary angle-closure, anterior segment optical coherence tomography

## Abstract

**Purpose:**

This study aimed to compare anterior segment parameters pre-and postoperatively in acute primary angle closure (APAC) and fellow primary angle closure suspect (PACS) eyes using anterior segment optical coherence tomography (AS-OCT) and evaluate the clinical effectiveness of cataract extraction in the treatment of APAC and fellow PACS eyes.

**Methods:**

Quantitative measurements of various parameters, including anterior chamber depth (ACD), anterior chamber volume (ACV), lens vault (LV), iridocorneal angle contact index (ITCI), iris thickness (IT), iris volume (IV), and iris curvature (IC), were obtained using Tomey CASIA2 AS-OCT on 60 eyes from 30 patients (APAC eyes and their fellow PACS eyes) before and after surgery. Simultaneous analysis of the differences between the APAC eyes and fellow PACS eyes in these parameters, visual acuity (VA), and intraocular pressure (IOP) were performed.

**Results:**

After surgery, both the APAC eyes and fellow PACS eyes (a total of 60 eyes) showed a significant increase in ACD and ACV, compared to preoperative measurements. Furthermore, LV and ITCI significantly decreased postoperatively. In the PACS group, IC significantly decreased postoperatively, while there was no statistically significant difference in the APAC group. In the APAC group, there was a significant decrease in IOP and improvement in VA at 1 day, 1 week, and the final follow-up compared to preoperative levels. The IOP values in the PACS group were within the normal range across various time points. VA in the PACS group showed significant improvement at 1 week postoperatively and at the final follow-up compared to preoperative levels. Significant differences of VA were observed in the initial, preoperative, first postoperative day, first postoperative week, and final follow-up, with better outcomes observed in the PACS group compared to the APAC group.

**Conclusion:**

Lens extraction surgery can significantly improve anterior segment crowding in APAC and PACS eyes. For APAC eyes, combined cataract extraction with intraocular lens implantation and gonioscopy-assisted goniosynechialysis under direct visualization is feasible and safe. Further, in the fellow PACS eye of APAC patients with either significant or mild cataracts, phacoemulsification can maintain or improve preoperative visual acuity to varying degrees and stabilize IOP.

## Introduction

Glaucoma is the second leading cause of blindness worldwide, and primary angle-closure (PAC) disease (PACD) is the most common type of glaucoma in China (1). Acute primary angle closure (APAC) is a distinct form of PACD and is considered an ophthalmic emergency. It is characterized by the sudden closure of the anterior chamber angle, followed by a rapid increase in intraocular pressure (IOP). Without timely treatment, APAC can rapidly lead to blindness, and once blindness occurs, there are currently no effective vision restoration methods available (2). In recent years, the development and continuous improvement of anterior segment optical coherence tomography (AS-OCT) have made it possible to obtain highly detailed imaging of the anterior chamber structures. These images have revealed the significant role of the lens in the angle closure development. Cataract extraction surgery is increasingly being employed in treating PACD at different stages, and it has become one of the main therapeutic approaches for managing PACD.

Laser peripheral iridotomy (LPI) has always been the recommended initial treatment for PAC and PAC suspect (PACS) due to its effectiveness in alleviating pupillary block, the main underlying mechanism of angle closure (2). However, LPI alone cannot open all narrowed angles in patients with PACS (3). Even after undergoing LPI, PACS eyes can still develop peripheral anterior synechiae (PAS) and/or increased IOP (4, 5). In patients with APAC, LPI is also not optimal for long-term IOP control, as 58% of patients experience increased IOP within 5 years after the initial attack (4). Lens extraction surgery for treating PACD is an increasingly interesting topic, yet consensus on its efficacy remains elusive. However, a significant amount of research on this topic mainly focuses on PAC glaucoma (PACG) or unclearly staged PAC cases (6–9). Therefore, this study explored the clinical efficacy of phacoemulsification lens extraction surgery for treating eyes with APAC and fellow eyes with PACS.

Anterior segment optical coherence tomography has been used for the evaluation of PACD parameters in recent years, but most studies have focused on anterior angle parameters such as AOD, TIA, and TISA. while there is relatively little research on iris parameters. We compared the preoperative and postoperative iris parameters, including iris trabecular contact index (ITCI), iris thickness (IT), iris volume (IV), and iris curvature (IC), and iris area (IA), of the APAC eyes and fellow PACS eyes using AS-OCT.

This study aimed to evaluate changes in anterior segment parameters, visual acuity (VA) and IOP before and after lens extraction surgery in patients with APAC and fellow PACS eyes. The findings of this study will provide insights into selecting clinical treatment strategies for patients with PACD, especially for those with one eye affected by APAC and the other eye affected by PACS.

## Methods

### Study participants

This retrospective study adheres to the principles of the Helsinki Declaration and has been approved by the Ethics Committee of Shandong Lunan Eye Hospital, Shandong Province. The analyzed data includes patients who received consecutive treatment in the glaucoma department of Shandong Lunan Eye Hospital, Shandong Province, from November 2022 to May 2023. A total of 30 patients were included in the study, with one eye diagnosed with APAC and the fellow eye diagnosed with PACS. The diagnosis of APAC and PACS was based on the diagnostic criteria of the International Society of Geographical and Epidemiological Ophthalmology (ISGEO) (10). All 30 patients underwent bilateral lens extraction with intraocular lens (IOL) implantation. In APAC eyes, additional gonioscopy-assisted direct visualization of angle separation was performed. All patients were over 50 years of age and had varying degrees of cataracts.

Exclusion criteria:

1. Patients with simultaneous acute attacks in both eyes.
2. Patients with a history of recurrent minor attacks and evident optic nerve damage.
3. Patients who had previously undergone any laser or intraocular surgery (including LPI, laser iridoplasty, trabeculectomy, and any other glaucoma-related treatments or intraocular surgeries).
4. Patients with known eye diseases that affected the anatomy of the anterior segment, such as ciliary or iris cysts, history of trauma, or use of local medications that affect iris configuration.
5. Patients with a history of any other intraocular diseases or comorbidities such as uveitis, diabetic retinopathy, macular degeneration, and retinitis pigmentosa.

### Ophthalmic examinations

All patients underwent baseline ophthalmic examinations during their initial visit. These examinations included VA (LogMAR), iCare tonometry for IOP measurement, slit-lamp biomicroscopy examination, ocular B-scan ultrasound, undilated fundus examination, anterior chamber angle examination, Ultrasound Biomicroscopy (UBM), and AS-OCT. A preoperative assessment of IOL power was performed using the Master700. VA, IOP, and slit-lamp biomicroscopy examinations were conducted on postoperative day 1, week 1, months 1 and 3. Furthermore, the AS-OCT examination was repeated at the third month postoperative visit.

### AS-OCT image acquisition and parameter measurement

All AS-OCT (Tomey CASIA 2, Tomey Corporation, Nagoya, Japan) examinations were performed for all patients pre-and post-operatively by the same experienced ophthalmic technician in a dark room environment. The initial AS-OCT examination of both eyes was conducted before the initiation of drug treatment in patients with acute attacks. A 1,310-nm swept-source laser wavelength with a frequency of 0.3 s was used in the CASIA 2 AS-OCT. It performed continuous scanning in the “anterior chamber angle” mode. The acquired images were analyzed using the software provided by the manufacturer. The built-in software automatically analyzed the anterior segment structures and provided measurement results after identifying the scleral spur (SS). The SS was defined as the point of curvature change where the sclera protrudes inward at the observed angle-scleral junction. A trained technician marked the SS without prior knowledge of anterior chamber angle grading. The analyzed parameters include the ITCI, defined as the percentage of iris trabecular contact area relative to the total measured area. Other parameters included anterior chamber volume (ACV), anterior chamber depth (ACD), anterior chamber width (ACW), anterior chamber area (ACA), and lens vault (LV), defined as the distance between the intersection of the perpendicular bisector of the SS connection and the midpoint of the SS connection with the lens, lens thickness (LT), IA, IV, IC, and IT at 750/2,000 microns (IT750/2000).

### Management of acute angle-closure glaucoma

The standard protocol involved initial management with topical and systemic medications to provide initial relief from acute angle-closure (AAC) attacks. If medication failed to alleviate the symptoms after 2 h, Laser peripheral iridoplasty was performed using a laser energy of 300–340 mW, spot diameter of 500 μm, and exposure time of 700 ms. If symptoms persisted and corneal edema prevented immediate lens extraction surgery, a low-dose cyclophotocoagulation procedure was administrated to lower IOP. All patients underwent ultrasonic phacoemulsification lens extraction surgery under good corneal transparency and controlled inflammatory response conditions.

### Surgical procedure

All surgeries were performed by a glaucoma specialist (Rumin Zhao) under topical anesthesia. Each patient underwent surgery on different times for both eyes. In the PACS group, transparent corneal incision phacoemulsification was performed, and a foldable IOL was implanted in the capsular bag. In the APAC group, combined direct visualization goniosynechialysis was performed in addition to the aforementioned procedure. Viscoelastic agents was injected into the anterior chamber, particularly in the angle area, to significantly deepen the peripheral anterior chamber. The patient’s head was elevated by 30°, the surgical microscope was tilted approximately 45°, and the ocular surgical gonioscopy lens (Ocular INSTR.SWAN JACOB GONIOPRISM, United States) was placed on the cornea for direct visualization of the angle structures. Through the main incision and side incision, an iris retractor was used to gently push the peripheral iris downward, and visible peripheral anterior synechiae (PAS) were released under direct visualization. The surgical endpoint was to achieve at least a visible scleral spur, with most cases showing visibility of the ciliary band and at least 180° of angle opening in the inferior quadrant. At the end of the surgery, the viscoelastic agent was removed from the anterior chamber thoroughly, and the incisions were watertight. Combined tension rings was implanted in cases with lens subluxation ranging from 90 to 180°. Postoperatively, all patients received topical antibiotics and steroids for 1 week, gradually tapering off according to clinical need within 4–6 weeks. In the APAC group, all antiglaucoma medications were discontinued postoperatively and reused as necessary.

### Statistical description

Statistical analysis was performed using the statistical software packages R (http://www.R-project.org, The R Foundation) and Free Statistics software version 1.7.1. Categorical variables were described as frequencies (%), while continuous variables were described as mean ± SD or median (range) if not normally distributed. Paired *t*-test was used to compare preoperative and postoperative parameters, a *p* value of <0.05 was considered statistically significant. The Bonferroni correction threshold was used to account for multiple comparisons and define statistical significance (0.05/14 = 0.0036 for primary analyses). The Kruskal–Wallis rank-sum test was also used to compare the visual acuity and IOP at different time points in each group.

## Results

### Description of basic characteristics of the study population

The average age of the 30 patients in the study was 66.6 ± 5.8 years, with 80% being females. Four patients had comorbid diabetes, and eight patients had comorbid hypertension. The BMI was 24.7 ± 3.2 (kg/mm^3^). The mean follow-up duration was 234.2 ± 59.6 days for APAC eyes and 222.2 ± 63.3 days for PACS eyes. The initial IOP for APAC eyes at diagnosis was 42.5 ± 16.2 mmHg. The preoperative IOP for APAC eyes was 19.0 ± 7.5 mmHg. The initial IOP for PACS eyes was 15.6 ± 9.8 mmHg. The preoperative IOP for PACS eyes was 14.4 ± 2.7 mmHg. The initial and preoperative VA for APAC eyes was 1.7 ± 1.1 and 1.0 ± 0.6, respectively. The initial VA and preoperative VA for PACS eyes was 0.3 ± 0.2. The axial length for both APAC and PACS eyes was 22.0 ± 0.7 mm. The preoperative ACD for APAC and PACS eyes was 1.7 ± 0.3 mm and 1.8 ± 0.3 mm, respectively. The postoperative ACD for APAC and PACS eyes was 3.4 ± 0.3 mm and 3.3 ± 0.6 mm, respectively. Table 1 summarized the various basic parameters.

**Table 1:** Demographics and characteristics of participants.

Variable		Total (*n* = 60)	APAC (*n* = 30)	PACS (*n* = 30)	*p*-value
Sex, *N* (%)	Female	--	24 (80.0)	24 (80.0)	--
	Male	--	6 (20.0)	6 (20.0)	--
Age (years)	--	--	66.6 ± 5.8	66.6 ± 5.8	--
Hypertension, *N* (%)	No	--	22 (73.3)	22 (73.3)	--
	Yes	--	8 (26.7)	8 (26.7)	--
Diabetes, *N* (%)	No	--	26 (86.7)	26 (86.7)	--
	Yes	--	4 (13.3)	4 (13.3)	--
BMI (kg/mm^3^)		24.7 ± 3.2	24.7 ± 3.2	24.7 ± 3.2	--
	≤0.1	3 (5.0)	0 (0)	3 (10)	
VA-first visit (logMAR)	>0.1, ≤0.3	11 (18.3)	0 (0)	11 (36.7)	
	>0.3, ≤1.0	24 (40.0)	9 (30)	15 (50)	
	>1.0	22 (36.7)	21 (70)	1 (3.3)	
VA-preoperation (logMAR)	>0.1, ≤0.3	14 (23.3)	3 (10)	11 (36.7)	
	>0.3, ≤1.0	28 (46.7)	13 (43.3)	15 (50)	
	>1.0	15 (25.0)	14 (46.7)	1 (3.3)	
IOP-first visit (mmHg)		29.1 ± 19.0	42.5 ± 16.2	15.6 ± 9.8	<0.001
IOP-preoperation (mmHg)		16.7 ± 6.1	19.0 ± 7.5	14.4 ± 2.7	0.002
Measures to reduce IOP	Medication alone	14 (23.3)	14 (46.7)	--	--
	Medication + LPIP	9 (15.0)	9 (30)	--	--
	Medication + LPIP + low-dose TSCPC	7 (11.7)	7 (23.3)	--	--
AL (mm)		22.0 ± 0.7	22.0 ± 0.7	22.0 ± 0.7	0.688
LT (mm)		5.1 ± 0.3	5.1 ± 0.3	5.1 ± 0.3	0.986
WTW (mm)		11.2 ± 0.5	11.2 ± 0.4	11.2 ± 0.6	0.534
Follow-up duration(day)		228.2 ± 61.2	234.2 ± 59.6	222.2 ± 63.3	0.452

APAC, Acute primary angle closure; PACS, Primary angle closure suspect; BMI, Body mass index; VA, Visual acuity; IOP, Intraocular pressure; LPIP, Laser peripheral iridoplasty; TSCPC, Transscleral cyclophotocoagulation; AL, Axial lengths; LT, Lens thickness; WTW, White-to-white corneal diameter.

### Comparison of preoperative and postoperative AS-OCT parameters in the APAC eyes and fellow PACS eyes

The postoperative ACV, ACA, and ACD significantly increased compared to the preoperative values in both groups. In addition, the LV and ITCI significantly decreased in both groups after the surgery. However, the differences in IV, ACW, peripheral IT, and IA between the preoperative and postoperative measurements were not statistically significant. In the PACS group, the postoperative IC significantly decreased compared to the preoperative measurement. In contrast, the difference in IC between the preoperative and postoperative measurements in the APAC group was not statistically significant (see Tables 2, 3).

**Table 2:** Comparison of preoperative and postoperative AS-OCT parameters in the APAC group.

Variables	Total (*n* = 60)	Preoperative (*n* = 30)	Postoperative (*n* = 30)	*p*-value
ACV (mm^3^)	101.204 ± 42.778	66.447 ± 12.763	135.961 ± 32.568	< 0.001
IV (mm^3^)	26.867 ± 6.515	26.464 ± 5.442	27.269 ± 7.510	0.506
ACD (mm)	2.526 ± 0.904	1.668 ± 0.269	3.384 ± 0.256	< 0.001
LV (mm)	0.066 ± 0.808	0.818 ± 0.324	−0.686 ± 0.231	< 0.001
ACW (mm)	10.637 ± 0.504	10.536 ± 0.542	10.739 ± 0.450	0.049
ACA (mm^3^)	17.349 ± 6.442	11.511 ± 3.128	23.186 ± 2.035	< 0.001
ITCI (%)	53.132 ± 41.479	90.397 ± 12.290	15.867 ± 21.820	< 0.001
Nasal IT750 (mm)	0.357 ± 0.140	0.362 ± 0.138	0.351 ± 0.144	0.776
Nasal IT2000 (mm)	0.339 ± 0.161	0.347 ± 0.149	0.331 ± 0.175	0.692
Nasal IA (mm^2^)	1.066 ± 0.403	1.135 ± 0.364	0.997 ± 0.433	0.155
Temporal IT750 (mm)	1.2 ± 6.4	2.0 ± 9.0	0.3 ± 0.1	0.826
Temporal IT2000 (mm)	0.3 ± 0.1	0.3 ± 0.1	0.3 ± 0.2	0.672
Temporal IA (mm^2^)	1.1 ± 0.4	1.2 ± 0.4	1.0 ± 0.4	0.062
Temporal IC	0.1 ± 0.1	0.2 ± 0.2	0.1 ± 0.1	0.062

ACV, Anterior chamber volume; IV, Iris volume; ACD, Anterior chamber depth; LV, Lens vault; ACW, Angle-closure distance; ACA, Anterior chamber area; ITCI, Iris trabecular contact index; IT, Iris thickness; IA, Iris area; IC, Iris curvature.

**Table 3:** Comparison of preoperative and postoperative AS-OCT parameters in the PACS group.

Variables	Total (*n* = 60)	Preoperative (*n* = 30)	Postoperative (*n* = 30)	*p*-value
ACV (mm^3^)	100.464 ± 41.017	65.885 ± 15.137	135.042 ± 26.830	< 0.001
IV (mm^3^)	30.519 ± 6.096	29.330 ± 6.636	31.708 ± 5.353	0.037
ACD (mm)	2.547 ± 0.865	1.802 ± 0.257	3.291 ± 0.558	< 0.001
LV (mm)	0.040 ± 0.831	0.841 ± 0.229	−0.760 ± 0.158	< 0.001
ACW (mm)	10.786 ± 0.465	10.710 ± 0.515	10.862 ± 0.404	0.086
ACA (mm^3^)	16.697 ± 6.271	11.332 ± 2.224	22.061 ± 3.941	< 0.001
ITC	25.863 ± 29.011	43.293 ± 27.058	8.433 ± 18.750	< 0.001
Nasal IT750 (mm)	0.338 ± 0.141	0.333 ± 0.120	0.344 ± 0.161	0.749
Nasal IT2000 (mm)	0.350 ± 0.173	0.344 ± 0.171	0.357 ± 0.177	0.764
Nasal IA (mm^2^)	1.236 ± 0.481	1.336 ± 0.388	1.136 ± 0.547	0.105
Temporal IT750 (mm)	0.322 ± 0.137	0.308 ± 0.130	0.336 ± 0.144	0.436
Temporal IT2000 (mm)	0.356 ± 0.129	0.361 ± 0.125	0.351 ± 0.135	0.781
Temporal IA (mm^2^)	1.305 ± 0.521	1.359 ± 0.540	1.252 ± 0.505	0.438
Temporal IC	0.148 ± 0.133	0.222 ± 0.140	0.073 ± 0.072	< 0.001

ACV, Anterior chamber volume; IV, Iris volume; ACD, Anterior chamber depth; LV, Lens vault; ACW, Angle-closure distance; ACA, Anterior chamber area; ITCI, Iris trabecular contact index; IT, Iris thickness; IA, Iris area; IC, Iris curvature.

### Comparison of VA and IOP between the APAC eyes and fellow PACS eyes

Differences in initial and preoperative IOP between the APAC eyes and fellow PACS eyes were statistically significant. However, the differences observed in postoperative IOP at various time points between the APAC eyes and fellow PACS eyes were nonsignificant. The initial, preoperative, first postoperative day, first postoperative week, and final follow-up VA showed significant differences between the APAC eyes and fellow PACS eyes. Compared to the APAC group, the PACS group consistently had better visual acuity (see Table 4).

**Table 4:** Comparison of IOP and visual acuity between the two groups.

Variables, Mean ± SD	Total (*n* = 60)	APAC (*n* = 30)	PACS (*n* = 30)	*p*-value
IOP-first visit	29.1 ± 19.0	42.5 ± 16.2	15.6 ± 9.8	< 0.001
IOP-preoperative	16.7 ± 6.1	19.0 ± 7.5	14.4 ± 2.7	0.002
IOP-first day after surgery	15.5 ± 4.1	15.8 ± 4.9	15.1 ± 3.3	0.398
IOP-1 week after surgery	14.5 ± 4.1	14.4 ± 4.9	14.6 ± 3.3	0.852
IOP-final follow up	14.6 ± 3.6	15.1 ± 4.0	14.1 ± 3.2	0.150
VA-first visit	1.0 ± 1.1	1.7 ± 1.1	0.3 ± 0.2	< 0.001
VA-preoperative	0.6 ± 0.6	1.0 ± 0.6	0.3 ± 0.2	< 0.001
VA-first day after surgery	0.4 ± 0.5	0.6 ± 0.6	0.1 ± 0.1	< 0.001
VA-1 week after surgery	0.3 ± 0.4	0.5 ± 0.5	0.1 ± 0.1	< 0.001
VA-final follow up	0.2 ± 0.3	0.4 ± 0.4	0.1 ± 0.1	< 0.001

APAC, Acute primary angle closure; PACS, Primary angle closure suspect; IOP, Intraocular pressure.

### Comparison of preoperative and postoperative VA and IOP within each group

**Figure 1:**
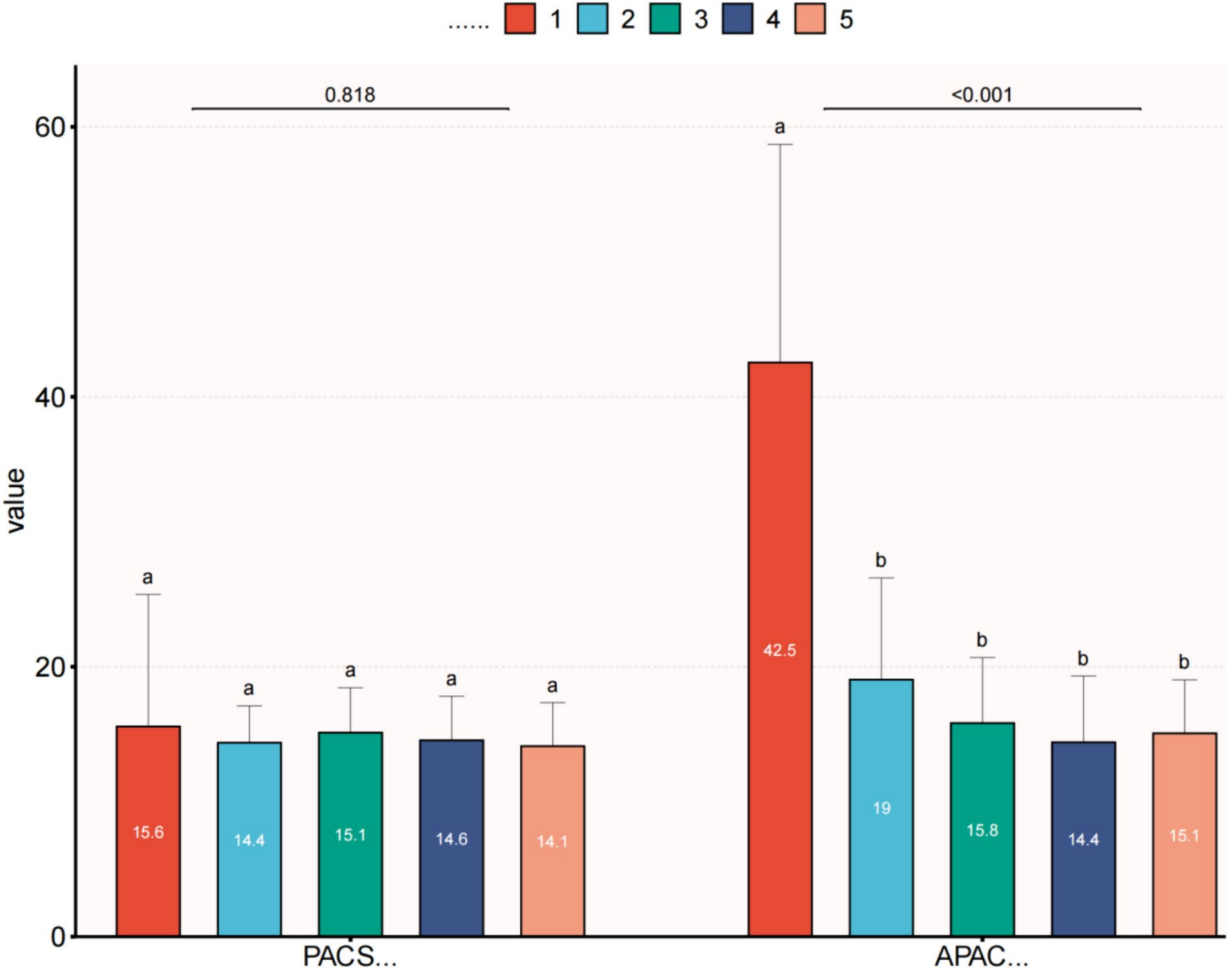
Comparison of preoperative and postoperative IOP within each group. (1) IOP—first visit; (2) IOP—preoperative; (3) IOP—first day after surgery; (4) IOP—1 week after surgery; and (5) IOP—final follow up. This image uses the letters A and B to show statistically significant differences between variables. For all variables with the same letter, the difference between the means is not statistically significant. If two variables have different letters, they are significantly different. APAC, Acute primary angle closure; PACS, Primary angle closure suspect; IOP, Intraocular pressure.

**Figure 2:**
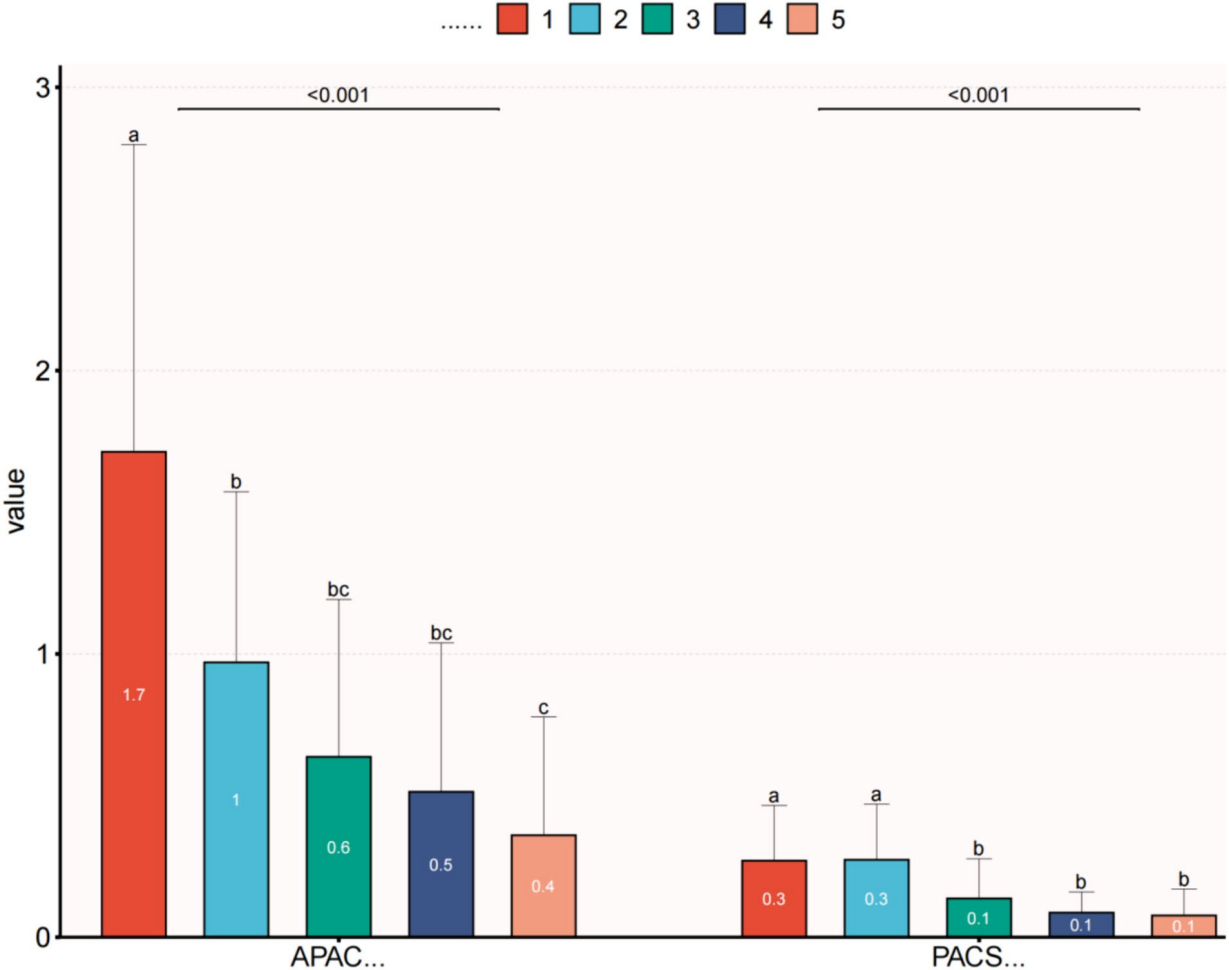
Comparison of preoperative and postoperative VA within each group. (1) VA—first visit; (2) VA—preoperative; (3) VA—first day after surgery; (4) VA—1 week after surgery; and (5) VA—final follow up. This image uses the letters A–C to show statistically significant differences between variables. For all variables with the same letter, the difference between the means is not statistically significant. If two variables have different letters, they are significantly different. APAC, Acute primary angle closure; PACS, Primary angle closure suspect; and VA, Visual acuity.

Within the APAC group, the average IOP significantly decreased to 15.8 ± 4.9 mmHg on the first day after surgery, 14.4 ± 4.9 mmHg at 1 week postoperatively, and 15.1 ± 4.0 mmHg at the final follow-up. All these time points showed significant differences compared to the preoperative IOP values. The VA in the APAC group improved to 0.6 ± 0.6 on the first day after surgery, 0.5 ± 0.5 at 1 week postoperatively, and 0.4 ± 0.4 at the final follow-up. All these time points showed significant differences compared to the preoperative VA. In the PACS group, there were no statistically significant differences in IOP at any postoperative time point compared to the preoperative IOP values. The VA at 1 week postoperatively and at the final follow-up significantly improved compared to the preoperative VA. However, there was no significant difference between the VA on the first day after surgery and preoperatively (see Tables 4, 5 and Figures 1, 2).

**Table 5:** Comparison of preoperative and postoperative AS-OCT parameters between the two groups.

		Preoperative	Postoperative	*p*-value
IOP (mmHg)	APAC	19.0 ± 7.5	15.1 ± 4.0	0.037
	PACS	14.370 ± 2.734	14.113 ± 3.223	0.259
	p	0.003	0.398	
VA	APAC	1.0 ± 0.6	0.4 ± 0.4	<0.001
	PACS	0.273 ± 0.196	0.077 ± 0.094	<0.001
	p	< 0.001	< 0.001	
ACD (mm)	APAC	1.668 ± 0.269	3.384 ± 0.256	<0.001
	PACS	1.802 ± 0.257	3.291 ± 0.558	<0.001
	p	0.052	0.409	

IOP, Intraocular pressure; VA, Visual acuity; ACD, Anterior chamber depth; APAC, Acute primary angle closure; PACS, Primary angle closure suspect.

## Discussion

In clinical practice, gonioscopy is considered the gold standard for diagnosing PACD. However, gonioscopy lacks the ability to quantitatively measure anterior chamber parameters. Ultrasound biomicroscopy (UBM) and AS-OCT can both be used for quantitative measurement of anterior chamber parameters (11). For more than 15 years, UBM has been utilized for this exact reason (12–14). With regard to narrow angles and other anterior chamber disorders, it offers a helpful diagnostic tool (15). Although UBM offers benefits, it also has certain drawbacks (16, 17). First of all, the patient may experience discomfort during this operation, which involves reclining supine and placing an eyecup between the lids. Some patients are not well-tolerated by this procedure. There are several potential impairments for the patient when using the UBM for imaging, including infection and corneal scratches. Furthermore, supine posture for UBM testing can affect chamber structures, especially in eyes with narrow angles the lens may shift posteriorly. This could result in missing certain occludable angles. New AS-OCT imaging enable anterior chamber angle to be measured in a noncontact manner and provide precise spatial relationships of the anterior segment structures (18). Compared to standard gonioscopy and UBM, AS-OCT can offer useful quantitative and spatial information regarding dynamic changes of the angle configuration due to its faster scan speed. When it comes to anterior chamber angle measurements, Tanuj Dada et al. (19) found that AS-OCT offers data that is comparable to that of UBM. Therefore, AS-OCT is widely used to obtain high-resolution anterior chamber images (20). It has evolved through several generations of innovation, from time-domain OCT (TD-OCT) to spectral-domain OCT (SD-OCT) and swept-source OCT (SS-OCT). CASIA 2 SS-AS-OCT, in particular, offers high scanning speed and provides higher depth sensitivity and resolution images. This enables the acquisition of parameter data from the cornea to the lens within just a few seconds.

This study found that for patients with PACS and APAC eyes, postoperative measurements of ACV, ACA, and ACD significantly increased compared to preoperative values, while LV and ITCI decreased significantly. However, the differences in IV, ACW, IT, and IA were not statistically significant because the surgery involved removing a thicker lens and replacing it with a thinner artificial lens, while the surgery itself had a minimal impact on iris tissue parameters other than IC. For the APAC group, postoperative IC changes were not significant compared to preoperative values. This may be because, during an acute attack of AAG, IOP increases suddenly, resulting in the closure of the angle and obstruction of aqueous humor outflow. Consequently, both anterior and posterior chamber pressures increase, leading to flattening of the iris and a decrease in IC. Therefore, there was no significant difference compared to postoperative values. On the other hand, the PACS group showed a certain degree of pupillary block and larger IC. The significant decrease in IC after surgery compared to preoperative values was mainly attributed to the relief of pupillary block factors following the removal of the crystalline lens.

Additionally, we found that both APAC eyes and their fellow PACS eyes showed varying degrees of visual improvement and a significant decrease in IOP after lens extraction surgery. This was because the removal of the crystalline lens eliminated pupillary block and reduced the angle crowding caused by a thick and anteriorly positioned lens, leading to a deepening of the anterior chamber and widening of the angle, facilitating the outflow of aqueous humor through the trabecular meshwork, resulting in a decrease in IOP (21, 22). Moreover, the surgery involved replacing the opaque lens with a transparent artificial lens, which contributes to the improvement in visual acuity to varying degrees.

In clinical practice, it is not uncommon to observe that after experiencing uncomfortable symptoms such as eye pain, headaches, gastrointestinal issues, and permanent damage to VA caused by a sudden increase in IOP, most patients have higher expectations for the visual quality of their other eye. They also have a greater urgency and desire for a safer and more reassuring treatment approach. LPI is widely used for the prophylactic treatment of patients with PACS (23). However, the Early Aggressive Treatment of Primary Angle Closure Glaucoma (EAGLE) study reported that early lens extraction is more cost-effective than LPI in the treatment of primary angle-closure glaucoma (PACG). Furthermore, transparent lens extraction leads to a more significant widening of the anterior chamber angle in PAC eyes without cataracts compared to LPI; however, the removal of a transparent lens remains controversial (6, 24). In this study, we selected lens extraction surgery for the fellow PACS eyes (including mild cataracts) of patients who experienced an acute attack in one eye. Our study results showed that for PACS eyes, the preoperative VA was 0.273 ± 0.196, which significantly improved to 0.077 ± 0.094 postoperatively. The preoperative and postoperative IOP were 14.370 ± 2.734 mmHg and 14.113 ± 3.223 mmHg, respectively. Throughout the follow-up period, the IOP remained stable, the pupil maintained a normal state, and the VA was better compared to that in APAC eyes, with lower IOP levels. This suggests that after lens extraction surgery, the VA of PACS eyes can either be maintained or show varying degrees of improvement compared to preoperative levels, in the meantime maintaining stable IOP. The removal of cataracts is beneficial for PACS eyes. However, it is important to note that this requires surgeons with extensive surgical experience and proficiency as a prerequisite. Adequate communication with the patient regarding the treatment options, including the purpose and significance of LPI and lens extraction, follow-up requirements, and long-term visual benefits, is crucial before surgery. Therefore, we believe that appropriately expanding the indications for lens extraction in the fellow PACS eyes of patients with APAC may be more beneficial for their long-term visual quality.

For APAC eyes, although the treatment of APAC has been described extensively in the textbooks, using LPI alone may not be sufficient to control the condition, and consensus on long-term management is still nonexistent. Combined trabeculectomy has also been used to relieve APAC when conventional medical treatment is ineffective. However, due to the significant inflammatory response in APAC eyes, the success rate of trabeculectomy is low, and the incidence of complications is high. These complications primarily include shallow anterior chamber, malignant glaucoma, progression of cataracts, suprachoroidal hemorrhage, and intraocular inflammation (25). Therefore, trabeculectomy is not considered the optimal approach for alleviating APAC. In this study, during the postoperative follow-up period of 30 APAC eyes, except for two patients who required additional antihypertensive medication to achieve adequate IOP control, the remaining patients had stable IOP control. The VA improved from 1.0 ± 0.6 preoperatively to 0.4 ± 0.4 postoperatively. This indicates that for APAC eyes, combined cataract removal with IOL implantation and gonioscopy-assisted goniosynechialysis is beneficial for both IOP control and visual acuity improvement.

In recent years, some researchers have suggested using cataract surgery as a treatment modality for PACD (26, 27) because PACD eyes can be affected by lens thickening, leading to pupillary block and increased IOP (28). Furthermore, the lens position in PACD eyes is more anterior than that of normal eyes (29–31). Cataract extraction combined with IOL implantation can deepen the anterior chamber and push the iris root backward, which widens the angle. Combined with goniosynechialysis, it can reopen the previously closed angle, making it one of the surgical treatment options for PACD patients (31, 32). However, most of the research on this topic has focused on PACG or PAC without clear staging. This study is the first to analyze both APAC eyes and fellow PACS eyes of the same patient who underwent cataract extraction simultaneously.

This study also had certain limitations. First, the sample size is relatively small, and further studies with numerous participants are needed. Second, the follow-up period in this study was 3–10 months postoperatively, and longer-term follow-up is required to observe the long-term effects on patients. Lastly, the methods of IOP reduction were inconsistent among different participants in the APAC group during acute attacks, which may impact certain angle parameters postoperatively. Future studies should aim to increase the sample size and analyze the effects of different methods of IOP reduction to enrich the research findings further.

## Conclusion

In summary, AS-OCT has numerous advantages in assessing the angle in glaucoma patients. The visual acuity of PACS eyes after lens extraction surgery is significantly better than that of APAC eyes, and the IOP is significantly lower. The VA of PACS eyes can either be maintained or show varying degrees of improvement compared to preoperative levels. Therefore, we believe that appropriately expanding the indications for lens extraction in the fellow PACS eyes of APAC patients is feasible. For APAC eyes, the postoperative outcomes of cataract extraction combined with IOL implantation and gonioscopy-assisted goniosynechialysis are commendable.

## Data Availability

The raw data supporting the conclusions of this article will be made available by the authors, without undue reservation.
